# Substrate and Mg doping effects in GaAs nanowires

**DOI:** 10.3762/bjnano.8.212

**Published:** 2017-10-11

**Authors:** Perumal Kannappan, Nabiha Ben Sedrine, Jennifer P Teixeira, Maria R Soares, Bruno P Falcão, Maria R Correia, Nestor Cifuentes, Emilson R Viana, Marcus V B Moreira, Geraldo M Ribeiro, Alfredo G de Oliveira, Juan C González, Joaquim P Leitão

**Affiliations:** 1 Departamento de Física & I3N, Universidade de Aveiro, Campus Universitário de Santiago, 3810-193 Aveiro, Portugal; 2 Crystal Growth Centre, Anna University, Chennai 600 025, India; 3 Present address: Department of Physics, Bannari Amman Institute of Technology, Sathyamangalam 638 401, India; 4 Laboratório Central de Análises, Universidade de Aveiro, 3810-193 Aveiro, Portugal; 5 Departamento de Física, Universidade Federal de Minas Gerais, 30123-970 Belo Horizonte, Minas Gerais, Brazil; 6 Departamento Acadêmico de Física-DAVIS, Universidade Tecnológica Federal do Paraná-UTFPR, Av. Sete de Setembro, 3165-Rebouças, ZIP 80230-901, Curitiba, PR, Brazil

**Keywords:** electronic structure, field effect transistors, GaAs nanowires, photoluminescence

## Abstract

Mg doping of GaAs nanowires has been established as a viable alternative to Be doping in order to achieve p-type electrical conductivity. Although reports on the optical properties are available, few reports exist about the physical properties of intermediate-to-high Mg doping in GaAs nanowires grown by molecular beam epitaxy (MBE) on GaAs(111)B and Si(111) substrates. In this work, we address this topic and present further understanding on the fundamental aspects. As the Mg doping was increased, structural and optical investigations revealed: i) a lower influence of the polytypic nature of the GaAs nanowires on their electronic structure; ii) a considerable reduction of the density of vertical nanowires, which is almost null for growth on Si(111); iii) the occurrence of a higher WZ phase fraction, in particular for growth on Si(111); iv) an increase of the activation energy to release the less bound carrier in the radiative state from nanowires grown on GaAs(111)B; and v) a higher influence of defects on the activation of nonradiative de-excitation channels in the case of nanowires only grown on Si(111). Back-gate field effect transistors were fabricated with individual nanowires and the p-type electrical conductivity was measured with free hole concentration ranging from 2.7 × 10^16^ cm^−3^ to 1.4 × 10^17^ cm^−3^. The estimated electrical mobility was in the range ≈0.3–39 cm^2^*/*Vs and the dominant scattering mechanism is ascribed to the WZ/ZB interfaces. Electrical and optical measurements showed a lower influence of the polytypic structure of the nanowires on their electronic structure. The involvement of Mg in one of the radiative transitions observed for growth on the Si(111) substrate is suggested.

## Introduction

In recent years, semiconductor nanowires have attracted a great deal of interest as building blocks for a new generation of electronic and optoelectronic devices, namely, batteries, biological and chemical sensors, thermoelectric devices, laser diodes, photo detectors, integrated photonic circuits, and solar cells [1–3]. Semiconductor nanowires have been a topic of intense research in the scope of third generation photovoltaic technology, with a predicted significant reduction of cost production [4]. Group III–V semiconductor nanowires are considered very promising materials for application in solar cells owing to their high absorption, direct bandgap, high carrier mobility and well-developed synthesis techniques [5–9]. Among the group III–V semiconductors, GaAs is one of the most intensively studied materials and has a suitable bandgap energy value for solar cells (1.519 eV for GaAs bulk at low temperature). Additionally, Ga is more abundant and less toxic than other elements (e.g., In and Cd, respectively) involved in other compounds commonly used in thin film-based solar cells like Cu(In,Ga)Se_2_ and CdTe. Bulk GaAs exhibits the zincblende (ZB) crystal structure, but when scaled down to the nanowire form, the occurrence of the wurtzite (WZ) crystalline phase is more prevalent due to a high surface-to-volume ratio and a low surface energy [10–12]. The WZ phase is more favorable for nanowires with small diameters, whereas for larger diameters, the ZB phase is favored [2]. In general, GaAs nanowires show a polytypic structure along the gowth axis that is characterized by the occurrence of a mixture of WZ and ZB phases [12–14]. This fact creates an unintentional bandgap that critically influences the optical and electrical properties of the nanowires [11–19]. In addition to the intentional doping, the distribution of crystalline phases and defects (like stacking faults and twin planes) along the nanowire length depends on the interplay between various growth parameters, namely temperature, effective V/III ratio during the growth and absolute pressure of the system [20–21]. Thus, the control of the crystalline phases in the nanowires is very important in order to reach the high mobilities expected for these low-dimensional structures [22].

Axial and radial approaches have been followed for the realization of p-n junctions in nanowire-based solar cells [5–6]. The control of the doping in GaAs is a fundamental issue. This is particularly true regarding the p-type doping if one intends to follow traditional architectures based on the use of n-type buffer layers, as is the case of thin film based solar cells [23–24]. Be has been the main choice to produce p-type GaAs layers and nanowires grown by molecular beam epitaxy (MBE) [25–28]. However, severe drawbacks like segregation at high concentration, a large diffusion coefficient prohibiting abrupt doping profiles and high toxicity have motivated research for alternatives [29–32]. Mg is another dopant impurity [25,33–38] used for p-type doping with a low diffusion coefficient, which has a solid solubility of 1 × 10^19^ cm^−3^ and a low sticking coefficient (10^−2^–10^−5^) in the substrate temperature range of 725–850 K [34]. The Mg atom occupies cationic sites in GaAs and creates an acceptor level in bulk GaAs with an ionization energy of 28 meV [33,35–36,39–41]. Only a few studies have focused on the optical and electrical properties of Mg-doped GaAs nanowires. So far, reports on the influence on the physical properties of intermediate-to-high Mg doping levels are scarce in the literature, with more relevance placed on the optical properties.

The envisaged applications of GaAs nanowires depend on the use of substrates with different compositions and orientations. The epitaxial growth of GaAs on Si substrates is of particular interest due to the possible integration with Si technology [42–43]. The free lateral surfaces of a nanowire allow efficient lateral stress relaxation [44], which is important to adjust lattice mismatched materials without the formation of a high density of structural defects [42,44]. However, the integration with Si requires overcoming a number of specific problems inherent to the dissimilarities in the physical properties of both materials, namely, lattice mismatch, significant differences in thermal expansion coefficients, and formation of antiphase domains due to the fact that GaAs can have polar planes (ZB or WZ) whereas Si has not [43,45]. Therefore, it is very important to study the effect introduced by the substrate on the physical properties of Mg-doped GaAs nanowires.

In this work, we study two samples containing Mg-doped nanowires, grown by MBE on GaAs(111)B and Si(111) substrates, with the same nominal Mg doping level. A thorough investigation of the morphological, structural, electrical and optical properties of the nanowires is presented. The X-ray diffraction (XRD) measurements suggest a polytypic structure. Electrical measurements performed on individual nanowire back-gate field effect transistors (FETs) allowed the estimation of the free hole concentration and the electrical mobility. The photoluminescence (PL) measurements revealed a few radiative transitions. The PL dependence on the excitation power and temperature was performed. The non-radiative de-excitation channels as well as the role of defects on the optical properties are discussed.

## Experimental

Mg-doped GaAs nanowires were grown on GaAs(111)B (sample A) and Si(111) (sample B) substrates by MBE in a Riber 2300 MBE reactor [14]. The nanowire growth was promoted through the Au-assisted vapor–liquid–solid growth mechanism by drop-coating the substrate with Au colloidal nanoparticles with average diameter of 5.0 ± 0.5 nm. The growth was performed at 615 °C for 90 min, under an As_4_ beam equivalent pressure (BEP) of 3.8 × 10^−5^ torr and Ga BEP of 7.2 × 10^−7^ torr. The Mg doping of the nanowires was achieved by keeping the Mg effusion cell at 240 °C. A nominal free hole concentration of 3 × 10^17^ cm^−3^ was estimated by Hall effect measurements performed in a GaAs epilayer, grown on a non Au-coated substrate, simultaneously with the nanowire samples. Despite the different factors that can influence the measured free hole concentration, we assume that it mainly reflects the Mg doping level. Thus, from now on we consider that a change in the free hole concentration is mainly due to a change in the Mg doping [46].

The morphology of the nanowires was studied by scanning electron microscopy (SEM) using high performance Schottky field emission HR-FESEM Hitachi SU-70 microscope equipped with in-lens secondary-electron and backscattered-electron detectors. The crystalline structure of GaAs nanowires was investigated by grazing incidence X-ray diffraction (GID) carried out on a PANalytical X’Pert MRD diffractometer using the Cu Kα_1_ radiation with a wavelength of λ = 1.540598 Å. The measurements were performed over the angular range 25–30°, with an increment of 0.02°, and for low incidence angles (ω = 0.1–2°).

**Figure 1:**
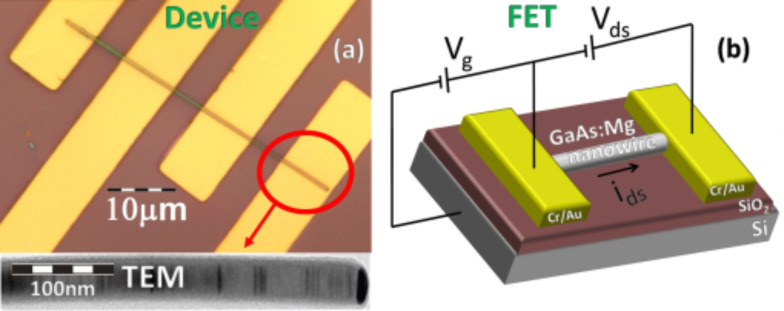
(a) Optical image of the back-gate GaAs:Mg nanowire FET device. The inset shows an illustrative TEM image of a Mg-doped GaAs nanowire showing the characteristic alternated WZ/BZ segments. (b) Schematic of the back-gated FET device.

For the transport measurements, back-gate FETs were assembled by mechanically transferring individual Mg-doped GaAs nanowires, grown on a GaAs(111)B substrate, with approximately constant diameters (≈190 nm) along the axis, onto a heavily doped Si substrate covered by a 300 nm thick SiO_2_ layer. Standard photolithography methods were used to define several contact lines, with a lateral separation of 3 to 9 μm on individual nanowires. Following this procedure, ohmic contacts with acceptable low contact resistance are obtained [19]. Figure 1a presents an optical image of a FET in which the nanowire and electrical contacts are observed. The TEM image at the bottom of Figure 1a shows the typical morphology of the GaAs nanowire, with an axial segmented structure created by the presence of WZ/ZB polytypism [14,17–19]. Figure 1b shows the scheme of the back-gated FET device and illustrates the gate voltage (*V*_g_) and source–drain voltage (*V*_ds_).

The optical properties were studied by measuring PL using a Bruker IFS 66v Fourier transform infrared (FTIR) spectrometer equipped with a liquid nitrogen cooled Ge detector. The samples were inserted in a helium flux cryostat that allowed temperature control in the range *T* = 5–300 K. The excitation wavelength was the 514.5 nm line of an Ar^+^ laser, focused on the sample with a long focal length lens, allowing an estimated laser spot diameter of 1 mm. The excitation power (*P*) was varied in the range ≈0.03–90 mW.

## Results

**Figure 2:**
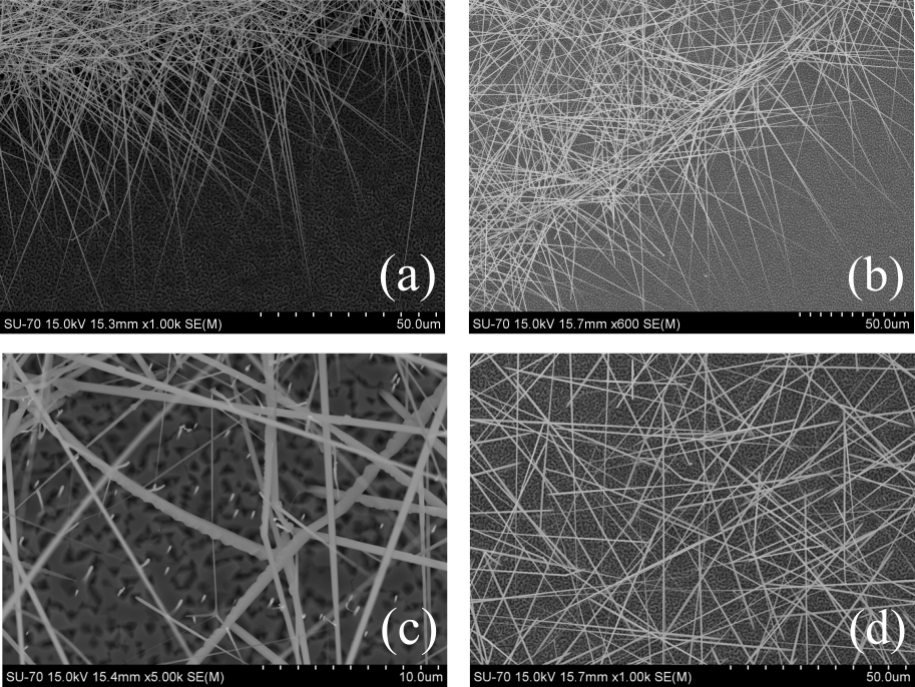
Scanning electron images of GaAs nanowires grown on GaAs(111)B (a,c) and on Si(111) (b,d) substrates. Images (a) and (b) show the border regions of bunches of nanowires, whereas (c) and (d) show the inner regions.

SEM images of tangled nanowires are presented in Figure 2: (a) and (b) show the border regions of bunches of nanowires in sample A and B, respectively, and (c) and (d) show equivalent images for inner regions. The lengths of the nanowires are up to several tens of micrometers whereas, for most of the nanowires, the diameter varies along the axis from a few hundreds of nanometers at the base to a few tens of nanometers at the tip. The orientation of non-vertical nanowires is apparently along particular directions, as can be seen in Figure 2a,b, in accordance with the common behavior reported in the literature [47–50]. The bright spots present in sample A (Figure 2c) correspond to vertical nanowires with diameters of a few tens of nanometers. Concerning sample B (Figure 2d), almost no vertical nanowires were observed. Thus, the increase of the Mg doping level caused a significant decrease of the density of vertical nanowires in both samples, as also observed in [14].

**Figure 3:**
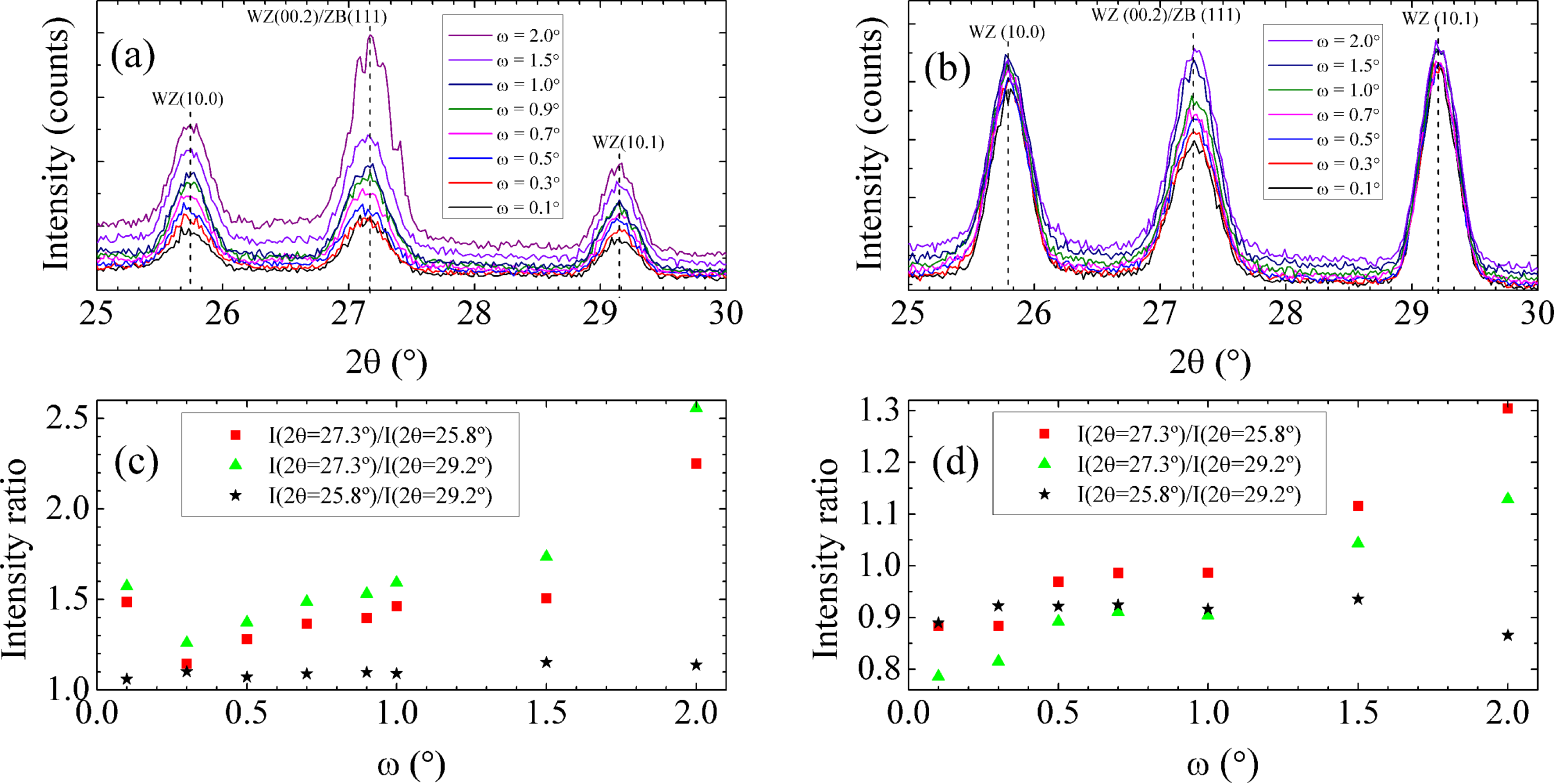
(a), (b) Grazing incidence X-ray diffraction diffractograms (ω/2θ) measured for samples A and B, respectively, obtained with different ω incidence angles. (c), (d) Ratios of the relative intensity of the peaks at 2θ = 25.8, 27.3 and 29.2° as a function of ω for samples A and B, respectively.

The investigation of the crystalline phases of the nanowires was performed by GID measurements. Previously, we have demonstrated that a decrease of the incidence angle ω reduces the contribution from the epilayer [14]. Similar measurements were performed here and are shown in Figure 3a,b for samples A and B, respectively. Regardless of the substrate, three reflection peaks with comparable relative intensities are observed in the range 25–30°. The peaks at ≈25.8 and 29.2° are ascribed to reflections in the WZ (10.0) and (10.1) planes, respectively, whereas the peak at ≈27.3° receives contributions of reflections from the WZ (00.2) and ZB (111) planes [14,51–52]. So far, the growth of the WZ crystalline phase was only observed in a reproducible way for low dimensional structures such as nanowires. For this reason, the WZ related peaks can only be assigned to WZ segments along the axis of the nanowires [10]. Concerning the peak at ≈27.3°, it can be related to contributions from reflections in WZ and ZB segments in the nanowires, but also from the ZB planes in the epilayer underneath the nanowires. The signal-to-noise ratio is lower for sample A in comparison with sample B. In order to quantify the change of the relative intensities of each peak as a function of ω, the three reflection peaks were fitted using Gaussian functions, which allowed a good fit to the experimental data (Supporting Information File 1). The estimated ratios of the relative intensities are shown in Figure 3c,d. It can be seen that by decreasing ω, the ratios *I*(2θ = 27.3°)/*I*(2θ = 25.8°) and *I*(2θ = 27.3°)/*I*(2θ = 29.2°) approach 1, showing a reduction in the relative intensity of the peak at ≈27.3°. This reduction is higher for sample A. We ascribe this behavior to the decrease of the contribution of the epilayer to the peak at ≈27.3° in both samples. However, that contribution cannot be excluded for the lower values of ω because no plateau is observed for the two above mentioned relative intensity ratios with the decrease of ω. In the case of sample A, the above mentioned relative intensity ratios for ω = 0.1° reveal an increase that does not follow the global trend of this sample. We relate this behavior with a misalignment that can lead to an increase of the contribution of GaAs epilayer underneath the nanowires. Consequently, the reduction for the peak at ≈27.3° is lower than it should be, explaining the behavior observed in Figure 3c. Regarding the WZ-related peaks, a reduction of the relative intensities is also observed in the case of sample A (see Figure 3a) and it should be related to the low amount of material in the WZ crystalline phase that is reached by the incident beam for each value of ω. For sample B, the relative intensities of the two WZ-related peaks are almost constant with ω and they are higher than the relative intensity of the peak at ≈27.3°. On the other hand, for both samples, the *I*(2θ = 25.8°)/*I*(2θ = 29.2°) ratio is approximately constant over all investigated values of ω and is relatively close to 1 (see Figure 3c,d). The above results are in agreement with the theoretical predictions stating that the intensities of the three WZ-related peaks are comparable [51] which suggests that for ω = 0.1°, the reflection peak at ≈27.3° is mainly related with the WZ phase present in the nanowires.

**Figure 4:**
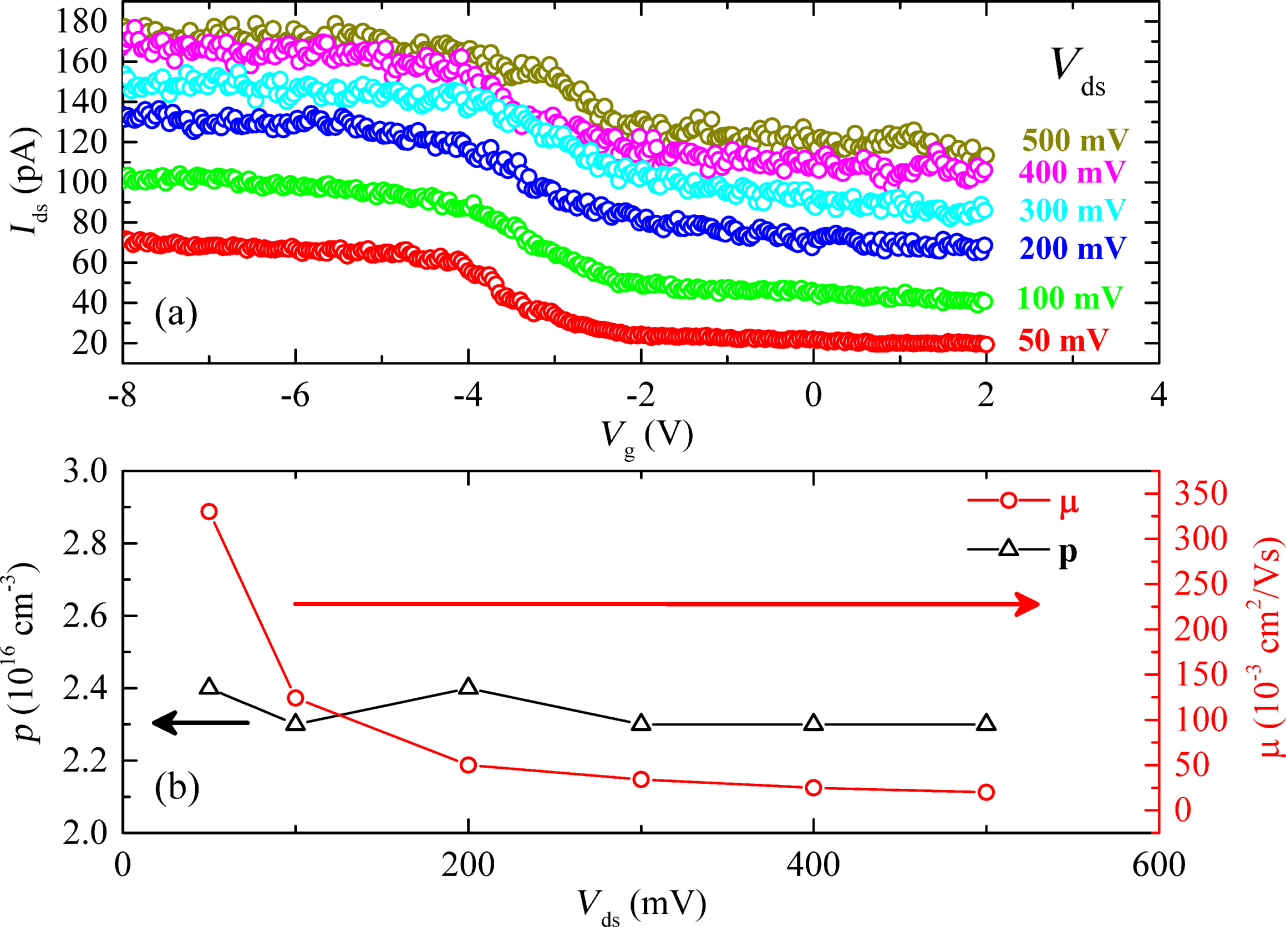
(a) p-type characteristic curves *I*_ds_–*V*_g_ of a FET based on the Mg-doped single GaAs nanowire #1 grown on GaAs(111)B substrate, as a function of *V*_ds_ and measured at 300 K. (b) Free carrier density and field mobility variation with *V*_ds_ in the FET nanowire.

Figure 1b shows the scheme of the back-gated FET device used for the electrical measurements performed on FETs of individual nanowires (#1, #2, #3) grown on the GaAs(111)B substrate (sample A). Figure 4a shows the source–drain current (*I*_ds_) vs gate voltage (*V*_g_) characteristics for a FET at 300 K, based on nanowire #1, measured as a function of the source–drain voltage (*V*_ds_) from 50 to 500 mV. All transfer curves show a p-type electrical conductivity for the Mg-doped single GaAs nanowire. For low values of *V*_ds_, the electrical field along the source–drain channel does not significantly affect the transport of the free carriers, allowing the extraction of the low field mobility and density of free carriers in the nanowire. As *V*_ds_ increases from 50 to 500 mV, the free carrier density in the channels remains approximately constant but the field mobility decreases significantly (Figure 4b). The mobility (μ) and density (*p*) of the free charge carriers in the FET channel can be estimated using the relation [53–56]:

[1]
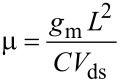


[2]
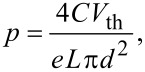


where the transconductance *g*_m_ = *dI*_ds_*/dV*_g_ and threshold voltage *V*_th_ are obtained from the slope and intercept of the linear region of the *I*_ds_–*V*_g_ curve, respectively.

For a back-gate nanowire FET, the capacitance (*C*) can be estimated considering a metallic cylinder-plane system from [19]:

[3]
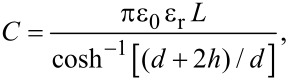


where *L* is the length of the FET channel, ε_0_ the vacuum permittivity, ε_r_ = 3.9 the relative dielectric constant of the SiO_2_ insulator layer, *h* = 300 nm the thickness of the SiO_2_ layer, and *d* the nanowire diameter.

From Equation 1–Equation 3 the field-effect mobility and charge carrier concentration have been calculated for three Mg-doped GaAs nanowires and the results are summarized in Table 1. As can be seen in Table 1, all Mg-doped GaAs nanowires have p-type electrical conductivity with the free hole concentration covering one order of magnitude, *p* = 2.7 × 10^16^, 3.8 × 10^16^ and 1.4 × 10^17^ cm^−3^, and the mobilities are μ = 0.33, 18.4 and 38.8 cm^2^/Vs, for the nanowires #1, #2 and #3, respectively. The free hole concentration values (Table 1) are lower than the nominal Mg concentrations obtained by Hall effect measurements on the epilayer. The difference between the free hole concentration and the nominal Mg concentration can be related to some degree of compensation or to the change of ionization energy of dopants close to the nanowire surface [57–59].

**Table 1:** Summary of the channel length (*L*), average nanowire diameter (*d*), threshold voltage (*V*_th_), transconductance (*g*_m_), hole mobility (μ) and free hole concentration (*p*), for three assembled FETs based on nanowires from sample A.

nanowire	*L*	*d*	*V*_ds_	*V*_th_	*g*_m_	μ	*p*
	(μm)	(nm)	(mV)	(V)	(S)	(cm^2^/Vs)	(cm^−3^)

#1	2.9	190	50	2.37	2.97 × 10^−11^	0.33	2.4 × 10^16^
#2	8.8	187	500	3.24	5.35 × 10^−9^	18.4	3.8 × 10^16^
#3	2.9	179	100	11.37	6.76 × 10^−9^	38.8	1.4 × 10^17^

**Figure 5:**
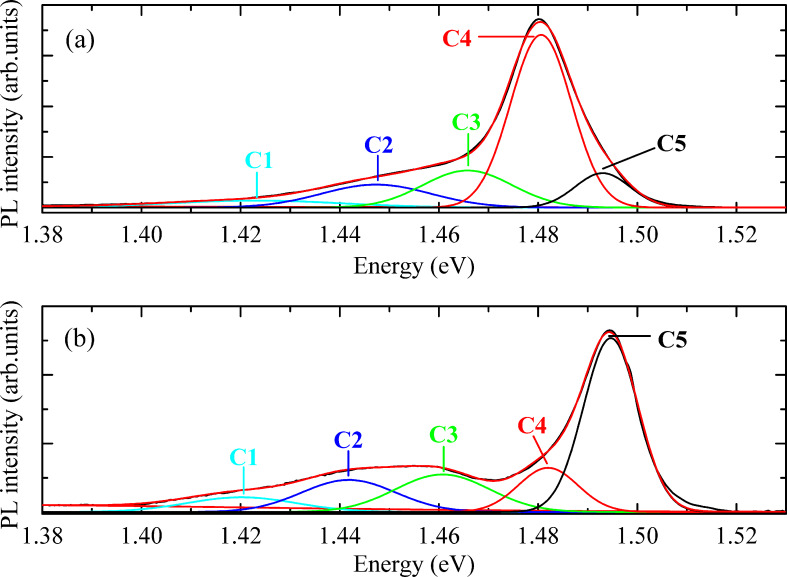
PL spectra of Mg-doped GaAs nanowires measured at ≈6 K under an excitation power of ≈27.7 mW, for (a) sample A and (b) sample B. The best-fit model with Gaussian components C# (# = 1, 2, …, 5) is illustrated.

The optical properties were investigated by PL measurements as a function of excitation power and temperature on a bunch of nanowires. In Supporting Information File 1, we present all PL spectra measured for both samples. It is important to mention that no significant influence from the epilayer is expected on the nanowire related luminescence, because no mensurable emission was registered when the laser was focused in a region of the sample’s surface without nanowires. Figure 5 shows the PL spectra for the two samples measured at ≈6 K and with an excitation power of ≈27.7 mW. Few radiative transitions are observed for both samples in the range ≈1.40–1.52 eV. The deconvolution of the PL spectra was made with Gaussian components assuming a model consisting in the lower number of components needed to describe the whole measured spectra for each sample. The peak positions of C4 and C5 are approximately the same in the two samples whereas their relative intensities are quite different. However, the peak position of C2 and C3 exhibit a slight blue-shift of ≈20 meV for sample A compared to sample B, showing comparable relative intensities. The luminescence in sample A is dominated by a transition at ≈1.481 eV (C4) whereas the PL spectrum in sample B is dominated by the component at ≈1.495 eV (C5). For the excitation power and temperature analyses of both samples, we will focus on discussing mainly the four Gaussian components at high energies, since C1 at ≈1.421 eV shows a low relative intensity which leads to significant uncertainty in the estimation of the peak energy and intensity.

**Figure 6:**
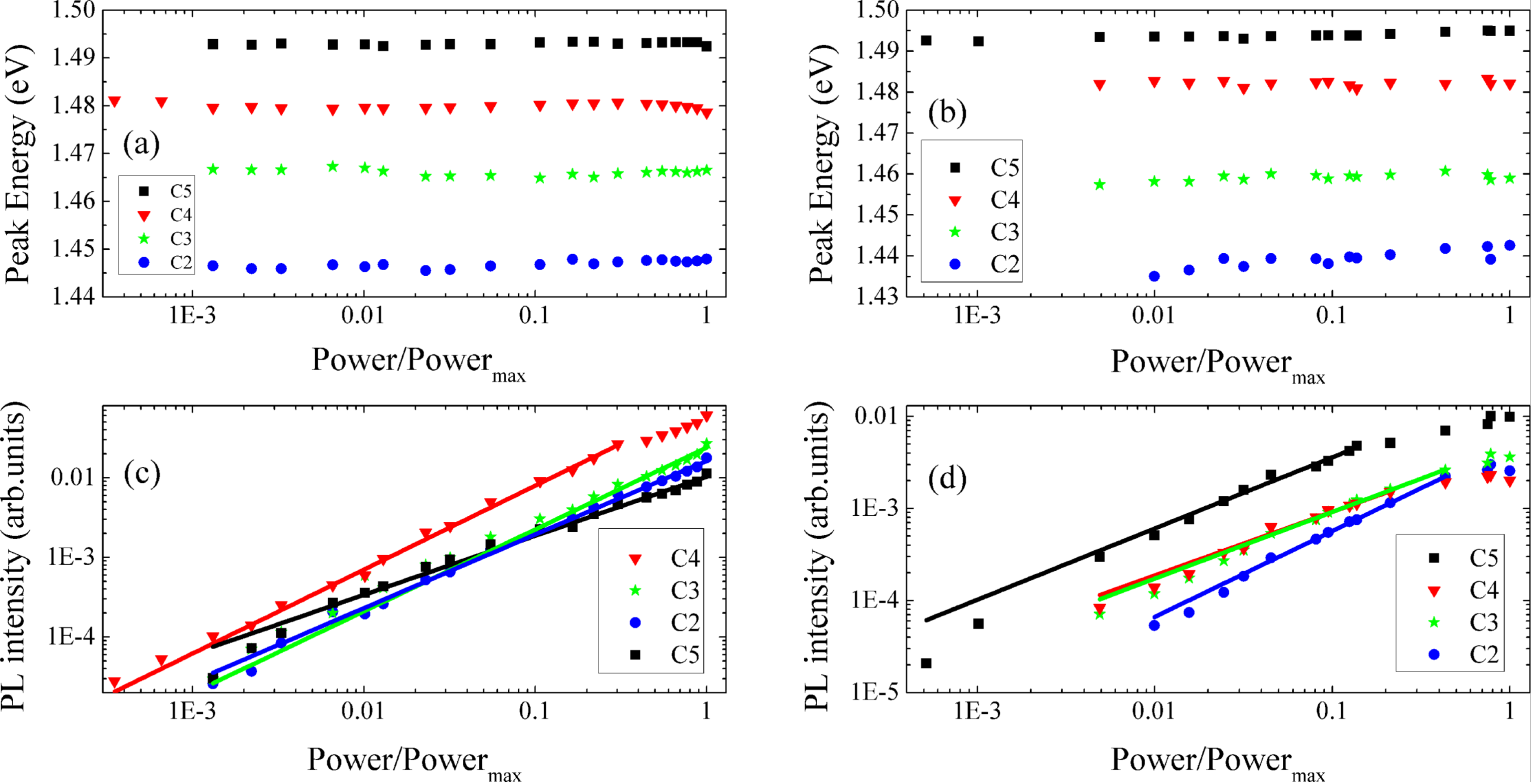
Dependence on the excitation power of the peak energy for (a) sample A and (b) sample B, and of the PL intensity for (c) sample A and (d) sample B. The lines in (c) and (d) are the fits of Equation 4 to the experimental points.

By increasing the excitation power, no significant shift of any of the four components is observed (see Figure 6a,b) whereas their relative intensities (*I*) increase. The dependence on the excitation power was performed through a wide range of approximately four orders of magnitude. The *I*(*P*) dependence can be described by [60]:

[4]
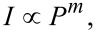


where *m* is an adjustable parameter. For *m >* 1 the radiative recombination is of excitonic nature, while for *m <* 1 some degree of localization of the charge carrier(s) has to be taken into account [60–61]. The fit of Equation 4 to the experimental points is shown in Figure 6c,d, and the estimated *m* values are summarized in Table 2. In the case of sample A and with the exception of C5, we obtained *m* values close to 1, whereas for sample B the values of *m* are lower than 1 for all components.

**Table 2:** Values of the peak energy (*E*_p_), measured at 6 K, of the Gaussian components for the two samples, and parameters estimated from the fitting of Equation 4 and Equation 5 to the experimental data.

Sample	Component	*E*_p_ (eV)	*m*	*c*_1_	*E*_1_ (meV)	*c*_2_	*E*_2_ (meV)	*c*_x_	*E*_x_ (meV)

A	C5	1.493 ± 0.001	0.74 ± 0.04	2.3 ± 0.1	2.8 ± 0.1	–	–	6.9 ± 3.8	76 ± 7
	C4	1.481 ± 0.001	1.05 ± 0.03	2.0 ± 0.1	5.1 ± 0.2	–	–	3.3 ± 0.8	63 ± 3
	C3	1.466 ± 0.001	1.03 ± 0.06	1.8 ± 0.1	4.4 ± 0.3	–	–	1.8 ± 0.6	41 ± 3
	C2	1.447 ± 0.001	0.92 ± 0.04	2.6 ± 0.1	5.2 ± 0.2	–	–	10.4 ± 5.2	64 ± 5
B	C5	1.495 ± 0.001	0.77 ± 0.03	7.7 ± 0.5	3.0 ± 0.1	67 ± 11	15 ± 1	–	–
	C4	1.482 ± 0.001	0.69 ± 0.03	105 ± 23	6.1 ± 0.3	–	–	–	–
	C3	1.461 ± 0.001	0.72 ± 0.02	0.18 ± 0.03	1.2 ± 0.1	10 ± 1	31 ± 2	–	–
	C2	1.442 ± 0.001	0.93 ± 0.01	3.2 ± 0.6	3.0 ± 0.4	–	–	820 ± 1942	39 ± 10

In order to better characterize the electronic energy level structure of the nanowires, a detailed investigation on the temperature-dependent PL is required. Before the presentation of the results, we will mention briefly the common temperature dependence of the bandgap in a semiconductor. As the temperature increases, the thermal expansion coefficient of the lattice and the electron–phonon interaction promote the broadening and the redshift of the energy levels, which leads to a reduction of the bandgap [46,62–63]. Among the several theoretical models available in the literature, one that probably better describes the temperature dependence of the bandgap in the whole temperature range for several semiconductors, namely for the ZB crystalline phase of GaAs, was proposed by Pässler [63]:

[6]
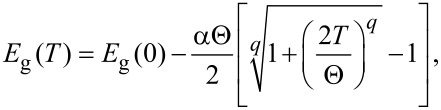


where *E*_g_(0) is the bandgap energy at 0 K, α is the *T*→∞ limit of *−dE*_g_(*T*)*/dT*, Θ is a parameter related with the Debye temperature, and *q* is an adjustable parameter.

**Figure 7:**
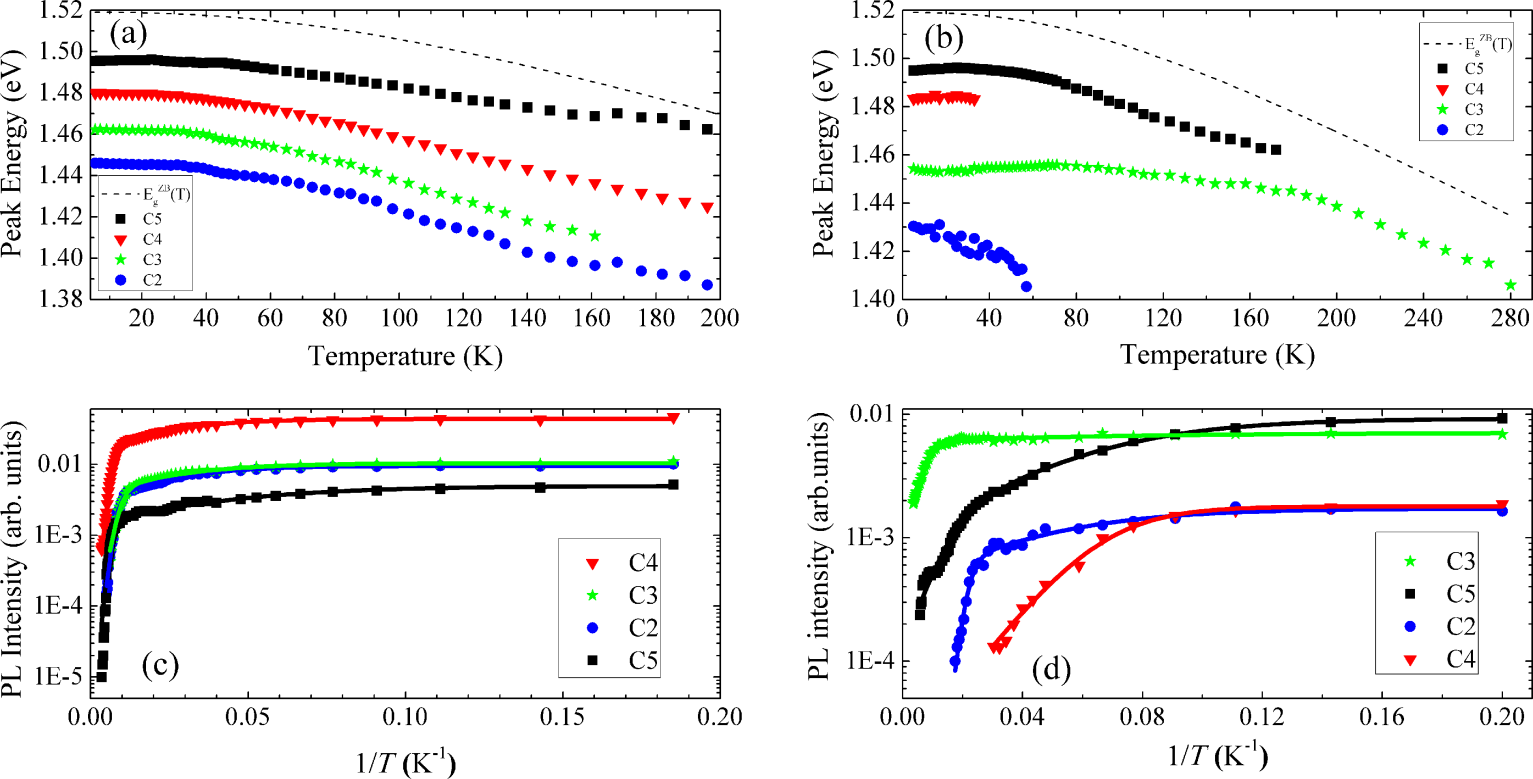
Dependence on the temperature of the peak energy for (a) sample A and (b) sample B, and of the PL intensity for (c) sample A and (d) sample B. The dashed lines in (a) and (b) represent the dependence on the temperature of 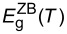 (Equation 6), using the parameters of Pässler for the ZB crystalline phase of GaAs [63]: *E*_g_(0) = 1.51909 eV, α = 0.4730 meVK^−1^, Θ = 225.6 K, *q* = 2.513. The lines in (c) and (d) are the fits of Equation 5 to the experimental points whose values are listed in Table 2.

In Figure 7, we present the results obtained for the dependence on temperature of the peak energy and PL intensity for the four components (C2–C5), measured under 60 and 50 mW for sample A and sample B, respectively. Additionally, in Figure 7a,b, the dashed lines present the expected behavior for 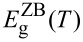 from Equation 6 using the parameters of Pässler for the ZB crystalline phase of GaAs [63]. Significant differences between the two samples must be pointed out. Firstly, by increasing the temperature, components C2, C3 and C4 of sample A follow roughly the behavior of 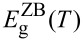, while C5 shows a lower slope for higher temperatures. The behavior of C5 can be explained by an increase of the uncertainty of the peak energy estimation in PL spectra having low signal-to-noise ratio. In the case of sample B, C5 follows roughly 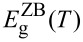. Regarding C3, no shift is observed for *T* 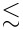 100 K and for *T* 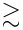 200 K the 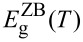 behavior is followed. On the other hand, the low number of experimental points for C2 and C4 hinders a deep discussion of their behavior. Secondly, the luminescence from samples A and B was observed up to 200 K and to room temperature, respectively. However, the behavior of each component is quite different. In the case of sample A, all components were observed until high temperature, which suggests the involvement of similar non-radiative de-excitation channels in the thermal quenching of these transitions. For sample B, C2 and C4 disappear at quite low temperatures (in the range 40–60 K), C5 quenches at ≈170 K, and C3 persists until room temperature. These results suggest a distinct role of the non-radiative de-excitation channels in the thermal quenching of these four components.

The non-radiative de-excitation channels were investigated considering an equation that takes into account the thermal redistribution of charge carriers between the radiative state and non-radiative discrete states (or a band) of higher energy [64]:

[5]
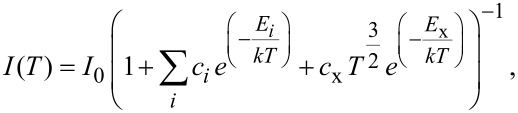


where *I*_0_ is the PL intensity at 0 K, the factors *c_i_* are parameters proportional to the ratio between the degeneracies of the high energy discrete state and the radiative state, the term *c*_x_*T^3/2^* accounts for the effective density of states of the band involved, and *c*_x_ is a fitting parameter. Different models were tested for each component of the two samples. The best one consists of the lower number of non-radiative de-excitation channels that allows us to well describe the thermal quenching of the PL. The resulting fitting parameters are listed in Table 2. We have found that for all components of sample A the model is the same and comprises two non-radiative de-excitation channels: one involving a discrete level (with activation energy *E*_1_), and one involving a band (with activation energy *E*_x_). The values of *E*_1_ are only a few meV, while the values of *E*_x_ are in the range 41–76 meV. The obtained *E*_x_ values mean that the PL intensity suffers a strong thermal quenching when *T* 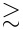 100 K, as mentioned above. Concerning sample B, only for C2 a similar model as the previous one was found (activation energies *E*_1_ = 3.0 meV and *E*_x_ = 39 meV for the discrete energy level and the band, respectively). However, the estimated error (10 meV) associated with the value of *E*_x_ is higher than the ones for the other estimated values (for all components, in both samples) due to the small number of experimental points in the high temperature region where the strongest PL thermal quenching occurs. For the other three components, the de-excitation channels only include discrete energy levels: two de-excitation channels for C3 (C5) with activation energies of *E*_1_ = 1.23 meV (3.0 meV) and *E*_2_ = 31 meV (15 meV), and only one de-excitation channel for C4 with *E*_1_ = 6.1 meV.

## Discussion

The nominal Mg doping (related to a free hole concentration *p* = 3 × 10^17^ cm^−3^) of the nanowires studied in this work represents an increment of the doping of approximately one order of magnitude in comparison with our previous work (*p* = 2 × 10^16^ cm^−3^) [14]. In this section, we will analyze the physical properties of the nanowires in order to discuss the influence of the substrate (GaAs(111)B and Si(111)), as well as the Mg doping. As will be discussed in the following, the results obtained here show a clear influence of the increase of the Mg doping level for both substrates, in particular for the Si(111) one.

Concerning the growth on GaAs(111)B substrate, the transport measurements confirmed the existence of a p-type electrical conductivity and demonstrated clearly the influence of the Mg doping level (3 × 10^17^ cm^−3^) on electrical properties of the nanowires. For a free-charge carrier density of *p* ≈ 10^16^ cm^−3^ in p-type bulk GaAs [65], mobilities in the order of 300–350 cm^2^/Vs are expected. These values are one to three orders of magnitude higher than those obtained here for Mg-doped p-type GaAs nanowires (μ ≈ 0.3–39 cm^2^/Vs). For two of the investigated nanowires, our values are in good agreement with a recent result (≈31 cm^2^/Vs) in p-type Si-doped GaAs nanowires, obtained by measuring the plasmon–phonon interactions using transmission Raman spectroscopy [59] and, on average, clearly higher than the reported value (0.417 cm^2^/Vs) obtained from similar measurements as ours [66]. The above increase of the mobility is quite relevant for optoelectronic applications. Another finding of this work is the increase of the mobility with the increase of the free hole concentration, in contrast to what is expected in a bulk monocrystalline material [67]. Thus, our results indicate that the free hole scattering mechanism in GaAs nanowires and in bulk GaAs are quite different. We wonder if the scattering on the surface of the nanowires could be responsible for these differences. It was reported that unpassivated GaAs NWs exhibit surface charge traps that induce a pinning of the Fermi level at the surface, especially for diameters below 100 nm [68]. However, if we compare the diameter of the three studied nanowires #1, #2 and #3, which were 190, 187 and 179 nm, respectively, the effect of surface scattering should be similar, which cannot explain the differences in the obtained mobility values. So, in our opinion, the scattering at the WZ/ZB interfaces along the nanowire’s axis is the dominant mechanism in Mg-doped GaAs nanowires, which is in accordance with the XRD results that show the occurrence of both crystalline phases in the nanowires. The highest mobility was obtained for the highest free hole concentration, suggesting that, in our case, the increase of free holes in the valence band progressively blurs the contribution of the polytypic nature of the nanowires on the electronic structure [61].

The obtained PL results reveal for the growth on GaAs(111)B substrate, a small influence of the Mg doping level on the peak energy of the main radiative transitions despite a similar shape of the emission [14]. The dominant radiative transition is still centered at ≈1.48 eV, but the other transitions show an apparent blueshift. However, concerning the excitation power dependence, the *m* values are near 1 for three (at lower energies) of the radiative transitions and lower than 1 for the transition at higher energy, which on average are higher than the *m* values found for the lower doping. In the first case, the values suggest a small localization of the charge carriers. In addition, we did not observe a meaningful shift of the emission when the power increases. These results are different from those obtained for lower Mg doping [14], where a blueshift and *m* values considerably lower than 1 were found. Actually, the experimental evidences in this work just suggest type-II radiative transitions at the WZ/ZB interface for the transition at higher energy. As the experimental evidence for the location of the carriers is now weaker, the possibility of transitions of type-I [69–70] should be considered despite the polytypism expected for the nanowires. These results are compatible with the increase of mobility observed for the raise of the free hole concentration. Both optical and electrical measurements reveal a clear influence of the doping level on the electronic structure of the nanowires which is reflected on the charge carriers dynamics. This is very important for the intended photovoltaic applications of these nanowires, in which the collection of charge carriers is a key issue.

On average, for growth on the GaAs(111)B substrate, the temperature dependence of the PL showed the thermal activation of non-radiative de-excitation channels of the same type found for lower Mg doping. Nevertheless, the activation energies in this work are clearly higher. This result suggests: i) a decrease of the possible confinement of charge carriers in the radiative states; ii) a non-significant change of the overall density of non-radiative defects as a consequence of the increase of the Mg doping. In the case of i), if the radiative transitions are related with recombination of charge carriers in each side of the WZ/ZB interfaces, larger segments of both phases should be present in the nanowires to allow a decrease of the confinement. The XRD results suggest a small increase of the WZ fraction in these nanowires but cannot clarify this question. We must note that PL is a technique that inspects a large quantity of nanowires simultaneously and the discussion of its results with the ones from local analyses like the ones provided, for example, by electron transmission microscopy, is always limited due to the lack of statistics provided by the latter. Regarding the redshift of the luminescence with the increase of temperature, the four radiative transitions follow roughly the behavior of 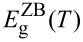. In summary, an overall consistent change of the electronic levels structure with the increase of the Mg doping is observed. The origin of the changes can be related to structural modifications induced by the higher doping.

For growth on the Si(111) substrate, the changes in structural and optical properties with the increase of the Mg doping and with the growth on GaAs(111)B substrate, are more evident. Starting with the XRD measurements, the WZ fraction is clearly higher than for the lower Mg doping or for the sample grown on GaAs(111)B substrate. Concerning PL, a significant change of the shape of the luminescence spectrum is observed, due to variations in the relative intensity of the transitions and the appearance of new ones at low energies. On the other hand, the estimated *m* values are lower than 1 for all transitions but no dependence on the excitation power of the PL peak energy was observed. For this sample, only the former results support type-II radiative transitions at the WZ/ZB interface [71–72]. In comparison with the sample for the growth on GaAs(111)B substrate, the thermal quenching of the luminescence showed a different behavior regarding the non-radiative de-excitation channels. The involvement of a band was observed only for the transition at 1.442 eV, whereas for the other three radiative transitions, only de-excitation channels related to discrete energy levels were identified. In addition, different temperatures of the PL extinction are observed for all transitions: for two of them the PL is quenched at 40–60 K, in accordance with the results for the lower Mg doping, whereas for the other two transitions, the luminescence is observed until much higher temperatures (even room temperature in the case of C3). We must note that for the growth on the Si(111) substrate, the activation energies (*E*_1_ and *E*_2_) for the de-excitation channels involving the discrete energy levels are lower than the estimated values (*E*_x_) for the complete release of the less bound charge carrier for the growth on GaAs(111)B substrate. Thus, the results suggest a higher influence of shallow non-radiative defects for the GaAs nanowires grown on a Si(111) substrate, which could be due to a higher density of defects as a result of the lattice mismatch between GaAs nanowires and the Si substrate.

In the case of the nanowires grown on a Si(111) substrate, the transition at 1.461 eV, observed up to room temperature, almost does not shift until ≈100 K and for *T* 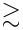 200 K it follows 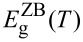. This behavior suggests the involvement of a relatively deep radiative state in a particular crystalline phase. Thus, the bandgap shrinkage induced by the increase of temperature will be more effective for high temperatures [46]. A higher localization of charge carriers in such a state is compatible with a value of *m* lower than 1 and non-radiative de-excitation channels involving discrete energy levels if a high density of non-radiative defects is assumed on that sample.

Concerning the hypothetical identification of the observed radiative transitions taking into account the reported associations from bulk GaAs [41], a few issues should be considered. Firstly, the data available in the literature is for ZB GaAs, due to the absence of growth of WZ in bulk. This fact hinders a comparison of similar point defects in both phases. Secondly, the polytypism involving single-phase segments with quite different thicknesses creates different levels of quantum confinement which is reflected in a spread of localization energies of charge carriers in the wells, thus affecting the photon energies of the radiative transitions. Thirdly, no experimental evidence exists concerning the possible presence of impurities, with the exception of Mg involved in the intentional doping. Fourthly, other effects such as strain [73–74] can influence the electronic level structure of the nanowires. From our results, in the case of the growth on the GaAs(111)B substrate, the estimated activation energy (*E*_x_) for the release of the less bound charge carrier in the radiative state is far from the ionization energy (28 meV) reported for Mg in the literature [41]. On the other hand, in the case of the growth on Si(111), and for the transition at 1.442 eV, the ionization energy is within the error of the estimated value for *E*_x_. Additionally, the estimated *m* value (0.93 ± 0.01) is compatible with the involvement of the Mg acceptor.

## Conclusion

The influence of the Mg doping and composition of the substrate (GaAs(111)B and Si(111)) were investigated for Mg-doped GaAs nanowires through morphological, structural, electrical and optical characterization. Both samples showed high densities of nanowires with lengths up to a few tens of micrometers, and diameters in the range of a few tens to hundreds of nanometers. All investigated nanowires have a p-type conductivity, with free hole concentration ranging from 2.7 × 10^16^ cm^−3^ to 1.4 × 10^17^ cm^−3^, and electrical mobility values in the range ≈0.3–39 cm^2^/Vs. The dominant scattering mechanism was ascribed to the WZ/ZB interfaces in accordance with XRD measurements which showed that the nanowires have both WZ and ZB crystalline phases.

With the increase of Mg doping, PL, XRD and SEM measurements revealed: i) a lower influence of the polytypic nature of the GaAs nanowires on their electronic structure; ii) a considerable decrease of the density of vertical nanowires, which is almost null for growth on Si(111); iii) the occurrence of a higher WZ phase fraction, in particular for growth on Si(111); iv) an increase of the activation energy to release the less bound carrier in the radiative state from nanowires grown on GaAs(111)B; and v) a higher influence of defects on the activation of non-radiative de-excitation channels in the case of nanowires grown on Si(111). Thus, the increase of the Mg doping has a significant effect on the properties of the nanowires grown on both types of substrates, with a particular relevance for the Si(111) case.

The experimental evidence for type-II radiative transitions is not as clear as in the case reported for a lower Mg doping. Indeed, the increase of the doping level suggests a waning of the constraints to the charge carriers dynamics created by the polytypic structure of the GaAs nanowires. This issue is very important for tuning the properties of nanowires in order to explore potential applications.

## Supporting Information

File 1 The supporting information illustrates the fitting model used in the evaluation of the relative intensities of the reflection peaks obtained in grazing incidence X-ray diffraction and presents all PL spectra measured using the excitation power and temperature dependencies for samples A and B.
