# Supplementary material for: Substrate and Mg doping effects in GaAs nanowires
Source: Beilstein J Nanotechnol. 2017 Oct 11;8:2126–38. doi: 10.3762/bjnano.8.212 (PMC5647700; doi:10.3762/bjnano.8.212)
Supplement: File 1 — The supporting information illustrates the fitting model used in the evaluation of the relative intensities of the reflection peaks obtained in grazing incidence X-ray diffraction and presents all PL spectra measured using the excitation power and temperature dependencies for samples A and B. [file Beilstein_J_Nanotechnol-08-2126-s001.pdf]

Supporting Information

for

Substrate and Mg doping effects in GaAs nanowires

Perumal Kannappan[1,2,3], Nabiha Ben Sedrine[1], Jennifer P. Teixeira[1], Maria R. Soares[4], Bruno P. Falcão[1], Maria R. Correia[1], Nestor Cifuentes[5], Emilson R. Viana[6], Marcus V. B. Moreira[5], Geraldo M. Ribeiro[5], Alfredo G. de Oliveira[5], Juan C. González[5], and Joaquim P. Leitão*[1]

Address: [1]Departamento de Física & I3N, Universidade de Aveiro, Campus Universitário de Santiago, 3810-193 Aveiro, Portugal, [2]Crystal Growth Centre, Anna University, Chennai 600 025, India, [3]Present address: Department of Physics, Bannari Amman Institute of Technology, Sathyamangalam 638 401, India, [4]Laboratório Central de Análises, Universidade de Aveiro, 3810-193 Aveiro, Portugal, [5]Departamento de Física, Universidade Federal de Minas Gerais, 30123-970 Belo Horizonte, Minas Gerais, Brazil, and [6]Departamento Acadêmico de Física DAFIS, Universidade Tecnológica Federal do Paraná - UTFPR, Av. Sete de Setembro, 3165-Rebouças, 80230-901, Curitiba, PR, Brazil

Email: Joaquim P. Leitão - joaquim.leitao@ua.pt

* Corresponding author

Fitting model used in the evaluation of the relative intensities of the reflection peaks obtained in grazing incidence X-ray diffraction and all PL spectra measured in the excitation power and temperature dependencies for samples A and B.

Figure S1 illustrates the fitting model used in the evaluation of the relative intensities of the reflection peaks obtained in grazing incidence X-ray diffraction.

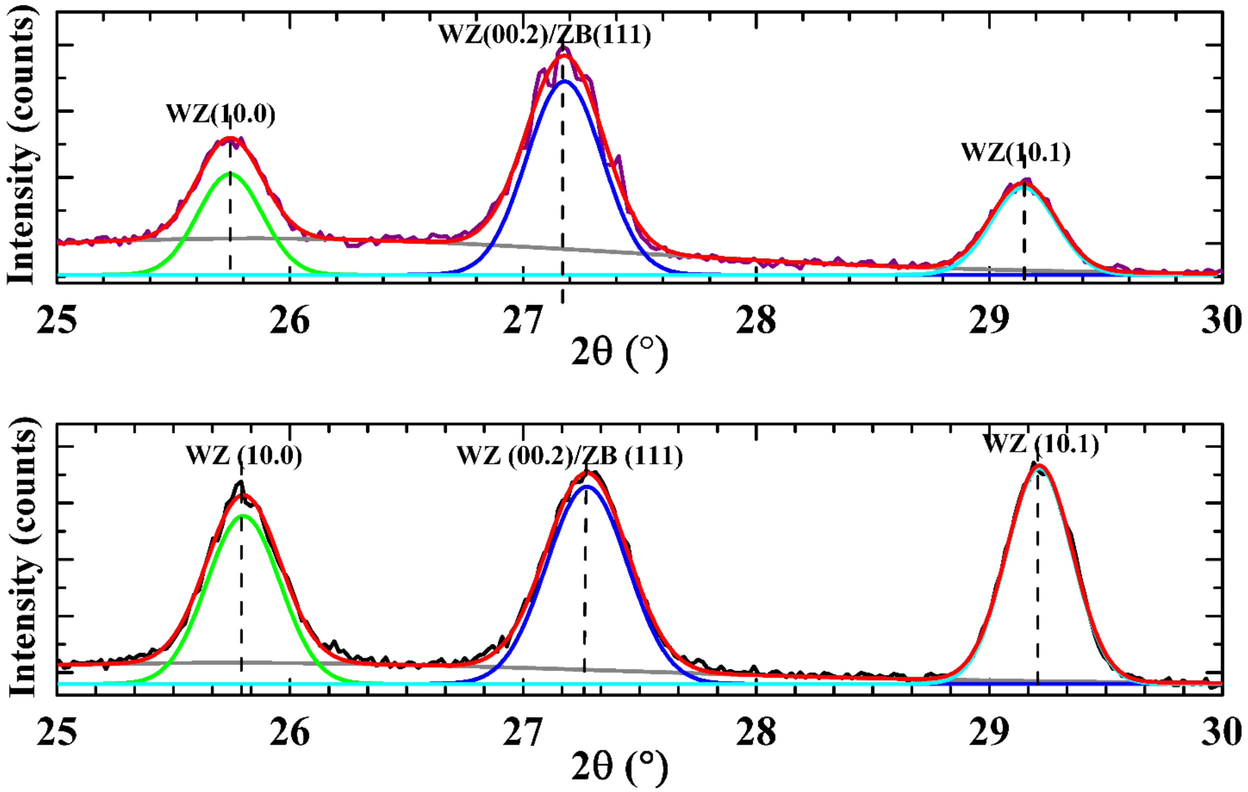

Figure S1: Grazing incidence X-ray diffraction diffractograms (ω/2θ) measured with an incidence angle of ω = 2, for (a) sample A and (b) sample B. The fitting model with Gaussian components to the observed reflection peaks is illustrated.

All PL spectra measured in the excitation power and temperature dependencies for both samples A and B are shown in Figure S2. The excitation power dependencies of both samples were performed at 5 K (Figure S2 (a) and (c)). The temperature dependencies for samples A and B were performed under excitation powers of 80 and 50 mW, respectively for Figure S2 (b) and (d).

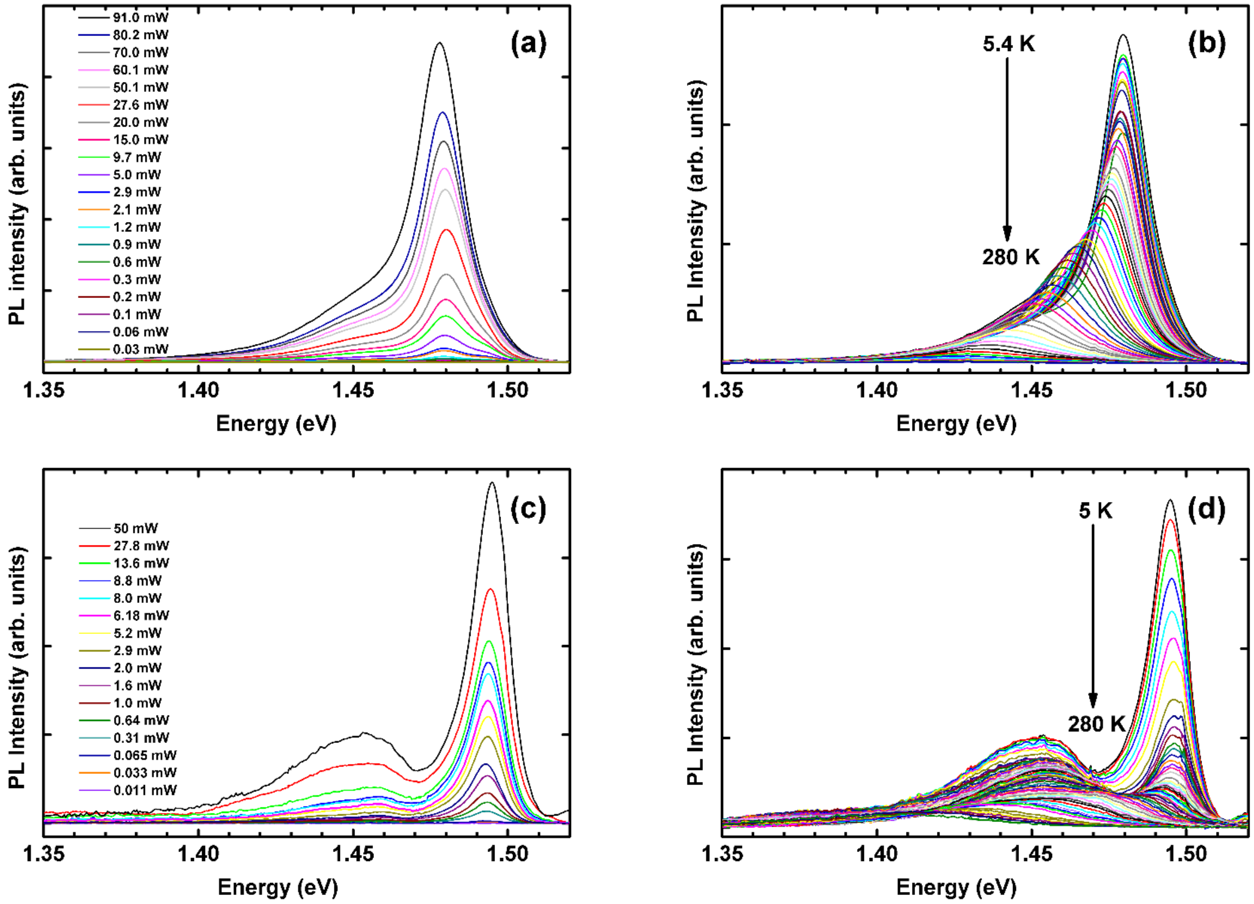

Figure S2: PL spectra measured in the excitation power and temperature dependencies for (a), (b) sample A; (c), (d) sample B. In (a) and (c) the temperature of the sample was 5 K. In (b) and (d) the excitation power was 80 and 50 mW, respectively.
